# Causal association between gut microbiomes and different types of aneurysms: a Mendelian randomization study

**DOI:** 10.3389/fmicb.2024.1267888

**Published:** 2024-04-10

**Authors:** Youjia Qiu, Yucheng Hou, Xingzhou Wei, Menghan Wang, Ziqian Yin, Minjia Xie, Aojie Duan, Chao Ma, Ke Si, Zhong Wang

**Affiliations:** ^1^Department of Neurosurgery & Brain and Nerve Research Laboratory, The First Affiliated Hospital of Soochow University, Suzhou, China; ^2^Department of Cardiovascular Surgery, The First Affiliated Hospital of Soochow University, Suzhou, China; ^3^Suzhou Medical College of Soochow University, Suzhou, China

**Keywords:** aneurysms, gut microbiome, abdominal aortic aneurysm, Mendelian randomization, genetic association

## Abstract

**Background:**

Previous studies suggests that gut microbiomes are associated with the formation and progression of aneurysms. However, the causal association between them remains unclear.

**Methods:**

A two-sample Mendelian randomization was conducted to investigate whether gut microbiomes have a causal effect on the risk of intracerebral aneurysm (IA), thoracic aortic aneurysm (TAA) and abdominal aortic aneurysm (AAA), and aortic aneurysm (AA). Single nucleotide polymorphisms (SNPs) smaller than the locus-wide significance level (1 × 10^−5^) were selected as instrumental variables. We used inverse-variance weighted (IVW) test as the primary method for the evaluation of causal association. MR-Egger, weighted median, weighted mode, and MR Pleiotropy Residual Sum and Outlier (MR-PRESSO) methods were conducted for sensitive analysis. The *p*-value was adjusted by the false discovery rate (FDR) which adjust the results of multiple comparisons, a *p <* 0.05 and *q <* 0.1 was considered a significant causal association. Additionally, a *p <* 0.05 and *q >* 0.1 was considered a suggestive causal effect. Additionally, reverse MR was also performed to exclude the possibility of reverse causality.

**Results:**

The phylum *Firmicutes* (OR = 0.62; 95% CI, 0.48–0.81), class *Lentisphaeria* (OR = 0.75; 95% CI, 0.62–0.89), and order *Victivallales* (OR = 0.75; 95% CI, 0.62–0.89) have a causal protective effect on the risk of AAA. Additionally, class *Verrucomicrobia*, class *Deltaproteobacteria*, order *Verrucomicrobiale*, family *Verrucomicrobiacea*, genus *Eubacterium rectale group*, genus *Akkermansia*, and genus *Clostridium innocuum group* were negatively associated with the risk of different types of aneurysms, whereas class *Negativicutes*, order *Selenomonadales*, and genus *Roseburia* had positive causal association with different types of aneurysms (*p <* 0.05; *q >* 0.1). Further sensitivity analysis validated the robustness of our MR results, and no reverse causality was found with these gut microbiomes (*p* > 0.05).

**Conclusion:**

Our MR analysis confirmed the causal association of specific gut microbiomes with AAA, and these microbiomes were considered as protective factors. Our result may provide novel insights and theoretical basis for the prevention of aneurysms through regulation of gut microbiomes.

## 1 Introduction

Aneurysm is a common cardiovascular disease characterized by gradual weakness of the localized arterial wall and permanent dilatation (Boese et al., 2017). It can affect arteries with different diameters in the human body, including the cerebral artery, thoracic aorta, abdominal aorta, and peripheral arteries (Breslin and Jewell, 1991). Of these, intracranial aneurysm (IA) is one of the common types, with a global prevalence of 3.2% (Vlak et al., 2011). It has been found that approximately 20% of IAs will eventually rupture, causing subarachnoid hemorrhage eventually, which results in a great financial burden around the world (Korja and Kaprio, 2016). Compared with IA, the incidences of thoracic aortic aneurysm (TAA) and abdominal aortic aneurysm (AAA) are even worse. Additionally, mortality in TAA and AAA was above 80% (Reimerink et al., 2013), and the financial burden caused by them was higher than that of IA (Dua et al., 2014; McClure et al., 2020). Currently, the primary treatment for aneurysm is surgical operation rather than preventing the occurrence and progression of it. No drug therapy has been proven effective in the prevention of aneurysms. Thus, it is important to explore new options that could be used in aneurysmal prevention.

Previous studies demonstrated that intervention in gut microbiota and its metabolites could not only reduce the development of aneurysms, but also prevent their rupture (Ling et al., 2022; Zou et al., 2022). Additionally, different types of gut microbiota are also associated with the formation of aneurysms (Xie et al., 2020; Zou et al., 2022). Xie et al. found that the gut microbiome was correlated with the diameter of AAA (Xie et al., 2020), while Shinohara et al. concluded that gut microbiota may participate in the accumulation of macrophages, which influenced the progression of aneurysm (Tian et al., 2022). However, considering the occult onset and poor prognosis of aneurysms, it is difficult to conduct clinical trials on patients with aneurysms. Ito et al. compared the gut microbiome in fecal samples between patients with IAs and healthy subjects, and concluded the dominance of *Firmicutes*, *Bacteroidetes*, *Proteobacteria*, *Fusobacteria*, and *Actinobacteria* in AAA group (Ito et al., 2023); while Nakayama et al. found an increased *Firmicutes*/*Bacteroidetes* ratio in aneurysmal wall compared to blood samples (Nakayama et al., 2022). It should be noted that these observational studies are limited by uncontrolled factors, such as age, environment, and dietary habits. Currently, only one prospective study with 61 samples compared the gut microbiome in fecal samples in unruptured and ruptured IAs (Kawabata et al., 2022). Although a prospective study could avoid the impact of uncontrolled factors, the limited sample sizes have constrained the assumption of causal association between aneurysm and gut microbiota.

Mendelian randomization (MR) is an analytic approach utilizing single nucleotide polymorphisms (SNPs) as instrumental variants to estimate the causal effect of a specific exposure on an outcome. Since the genetic variables are randomly allocated during meiosis, it could prevent the influence of reverse causality and confounding factors that has existed in observational studies (Smith and Ebrahim, 2003; Burgess et al., 2015). A previous study has explored the causal association between gut microbiota and cardiovascular disease (Zhang et al., 2022). However, a causal relation between gut microbiota and aneurysm has not been detected. In this study, we conducted a two-sample MR using recently published summary statistics of gut microbiota and aneurysm from genome-wide association study (GWAS). Clarifying this causal connection may help prevent the formation and progression of aneurysm.

## 2 Materials and methods

### 2.1 Ethics statement

Our study was a re-analysis of data already published in GWAS; all ethical approvals were obtained by the original GWAS authors. Thus, no additional ethical approval was required.

### 2.2 Study design

**Figure 1:**
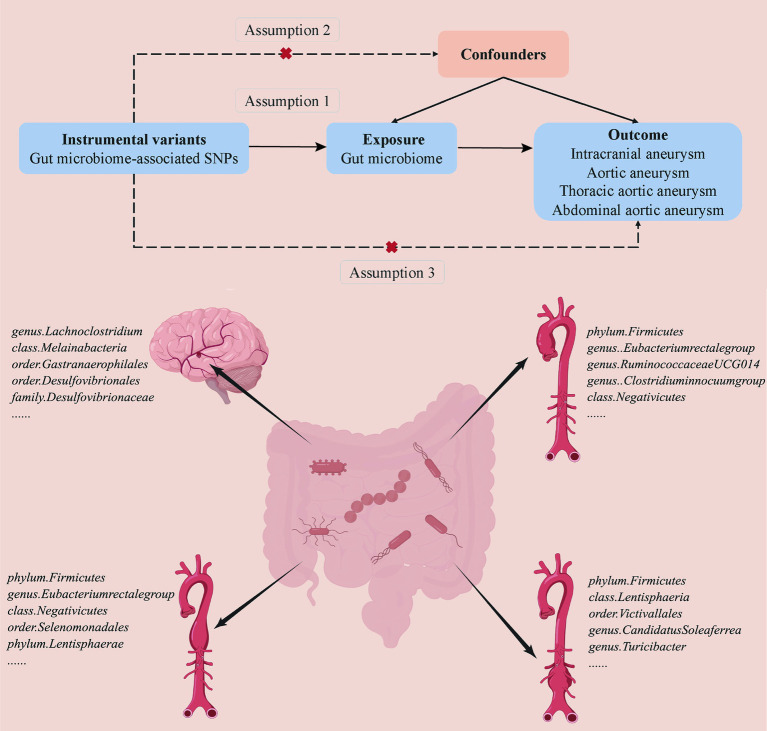
Overview of the suggestive causal association between gut microbiomes and different types of aneurysms.

A two-sample MR analysis was conducted to detect a casual association between the gut microbiome and aneurysms (Figure 1). Based on the strict selection criteria, Single nucleotide polymorphisms (SNPs) related to specific gut microbiomes served as instrumental variables (IVs). The MR design was based on the following assumptions: (1) genetic variants were robustly correlated with gut microbiota; (2) the genetic variants used were not associated with any confounding factors; and (3) the selected genetic variants affected the formation of aneurysm only through gut microbiota instead of via other pathways.

### 2.3 Selection of instrumental variants

In this MR study, we used the locus-wide significance threshold (*p <* 1 × 10^−5^) to obtain more relevant IVs (Jia et al., 2019; Lv et al., 2021; Guo et al., 2023; Li et al., 2023). Moreover, to ensure the independence of IVs, a clumping procedure with a stringent threshold (*r*^2^ *<* 0.001, kb = 10,000) was conducted. If linkage disequilibrium (LD) was present (*r*^2^ *>* 0.001), we used the SNPs with the lowest *p*-value when high LD existed (Zhang et al., 2022). When palindromic SNPs existed, we removed them to ensure the effect of SNPs on each gut microbiome corresponded to the same allele as the effect on the aneurysms. Finally, we calculated the F-statistic for SNPs to assess instrumental strength utilizing the following formula: F statistics = beta^2^/se^2^. SNPs with an F-statistic *<*10 would be removed (Pierce et al., 2011). When SNPs were unavailable in GWAS for exposure, we searched and selected proxy SNPs with an LD of at least *r*^2^ *>* 0.80 (Peters et al., 2021). Then we further searched the GWAS catalog (https://www.ebi.ac.uk/) to exclude SNPs that were potentially associated with confounding factors, such as blood pressure, hypertension, and diabetes (Staley et al., 2016; Karhunen et al., 2021).

### 2.4 GWAS data for gut microbiota

The genetic variants of gut microbiota were obtained from the GWAS of the MiBioGen consortium, which has the largest number of gut microbiome genetics research published to date (Kurilshikov et al., 2021). Our study enrolled 18,340 individuals, with 16S rRNA gene sequencing data from 24 cohorts in 11 countries, and all datasets were rarefied to 10,000 reads per sample. Then, taxonomic classification was conducted using direct taxonomic binning. The cutoff criteria for the study encompassed a minimum effective sample size of 3,000 individuals and participation in no fewer than three cohorts. In each cohort, the binary trait loci mapping (mbBTL) analysis included the taxa present in more than 10% of the samples and contained 211 taxa (131 genera, 35 families, 20 orders, 16 classes, and 9 phyla) with 122,110 variant sites (Kurilshikov et al., 2021).

### 2.5 GWAS data for aneurysms

The GWAS summary statistics of aneurysms were obtained from the FinnGen consortium (Kurki et al., 2023), which consisted of Finnish nationwide cohort and biobanks. Four main types of aneurysms diagnosed with 10th version of the International Classification of Diseases (ICD-10) were considered in our analysis, including IA, AAA, TAA, and aortic aneurysm (AA). The details of participants, statistical protocols, and genetic information are available at the FinnGen website (https://www.finngen.fi/en/). The data source of gut microbiomes and aneurysms was shown in Table 1.

**Table 1:** Overview of the summary data.

Characteristic	Resource	Sample size	Population	Data download
**Exposure**				
Gut microbiome	MiBioGen	14,306	European	http://www.mibiogen.org
		811	East Asian	
		1,097 114 481 1,531	Hispanic African American Middle Eastern Other admixed ancestry	
**Outcome**				
Intracranial aneurysm	FinnGen	1788 cases	European	https://r8.finngen.fi/
		312,768 controls		
Thoracic aortic aneurysm	FinnGen	7,321 cases	European	https://r8.finngen.fi/
		317,899 controls		
Abdominal aortic aneurysm	FinnGen	3,201 cases	European	https://r8.finngen.fi/
		317,899 controls		
Aortic aneurysm	FinnGen	7,603 cases	European	https://r8.finngen.fi/
		317,899 controls		

### 2.6 Statistical analysis

We used the inverse variance weighted (IVW) test as the principal analysis method, which combines each SNP-specific Wald estimate (SNP-outcome β divided by the SNP-exposure β) and calculates an overall estimate of the effect of the exposure on the outcome via a meta-analysis approach (Bowden et al., 2017). A fixed or random effect model was selected according to the existence of heterogeneity. When there was no horizontal pleiotropy, IVW could prevent the influence of confounding factors and achieve unbiased estimates (Xiang et al., 2021). Then we compared the results of IVW with other methods that were used for sensitive analysis, and the consistent direction of all methods indicated a higher confidence level of the evidence (Cai et al., 2022).

To evaluate the robustness of our findings, MR-Egger, weighted median, weight mode, and MR Pleiotropy Residual Sum and Outlier (MR-PRESSO) were performed as sensitivity analyses. MR-Egger regression is a meta regression of SNPs exposure association against SNPs outcome associations with a non-fixed y-axis intercept. If the intercept is equal to zero, there is no horizontal pleiotropy and the result is consistent with IVW (Bowden et al., 2015). This method can detect and correct for directional pleiotropy but the result might be influenced by outlying genetic variables (Xiang et al., 2021; Morris et al., 2022). The weighted median method can yield robust estimates of causality when there is 50% invalid IVs (Bowden et al., 2016). It shows greater detection of causality, less deviance, and a lower rate of type I error compared with MR-Egger when the instrument strength independent of the direct effect (InSIDE) hypothesis is violated (Hartwig et al., 2017). When the majority of similar individual instrument causal effect estimates comes from valid instruments, the weight mode method is valid even if IVs are not consistent with causal interference using the MR method (Hartwig et al., 2017; Ooi et al., 2019). The MR-PRESSO method was used to detect horizontal pleiotropy via a global test. It could also examine whether the causality estimation would change with the exclusion of outlying SNPs (*p <* 0.05) (Verbanck et al., 2018).

The heterogeneity was evaluated by Cochran’s *Q* test using the IVW method (the significance level was set to 0.05). In addition, the leave-one-out analysis was conducted to test whether stability of causality was strongly driven by an individual outlier. We also conducted reverse MR analysis on gut microbiomes that had causality association with aneurysms, and the method’s results were congruent with those of MR analyses (Verbanck et al., 2018).

The FUMA platform was used to facilitate functional annotation of the GWAS results, gene prioritization and interactive visualization to identify potential lead SNPs (Watanabe et al., 2017). To identify whether causal SNPs influence the expression of genes, we mapped lead SNPs to the closest (within a 1-Mb frame) genes (Islam et al., 2022). Based on genes mapped by lead SNPs from significant gut microbiota, we conducted an enrichment analysis that was matched to genes to further explore the biological role of gut microbiota that had a suggestive association (*p* < 0.05) with aneurysm. The annotation of functional gene ontology (GO) was retrieved from MSigDB v 7.0 (Subramanian et al., 2005). In addition, we examined the enrichment in 30 common body tissues of the Genotype-Tissue Expression (GTEx) data sets.

All the analyses were conducted using R software (Version 3.5.3). MR analysis of causal association between gut microbiomes and aneurysms was performed using the “TwoSampleMR” package. The MR-PRESSO analysis was performed using the “MR-PRESSO” package. The causal influence of suggestive gut microbiomes on risk of aneurysms was displayed the by R software “ggplot2” package. Odds ratio (OR) reflects the causal effect of gut microbiomes and aneurysms, and presents an increased risk of binary outcomes (aneurysms) per SD increase in abundance of gut microbiomes. The *p*-value was adjusted by the false discovery rate (FDR) which adjust the results of multiple comparisons (Li et al., 2022). A *p <* 0.05 and *q <* 0.1 was considered a significant causal association. Additionally, a *p <* 0.05 and *q >* 0.1 was considered a suggestive causal effect (Waters and Ley, 2019; Xiang et al., 2021).

## 3 Results

### 3.1 Selection of IVs

Overall, 503 SNPs were identified as final IVs for 56 gut microbiomes under the locus-wide significance threshold (*p <* 1 × 10^−5^). Specifically, there were 5 for phylum, 6 for class, 8 for order, 8 for family, and 30 for genus. For different types of aneurysms, we found that 229 SNPs with 24 gut microbiomes was associated with AA, 207 SNPs with 23 gut microbiomes for IA, 152 SNPs with 19 gut microbiomes for TAA, and 146 SNPs with 19 gut microbiomes for AAA. In addition, all F-statistics were more than 10, indicating there was no evidence of weak IV bias. The detailed information of SNPs (i.e., effect allele, other allele, beta, standard error, *p*-value and EAF) are shown in Supplementary Table S1.

### 3.2 MR analysis

**Figure 2:**
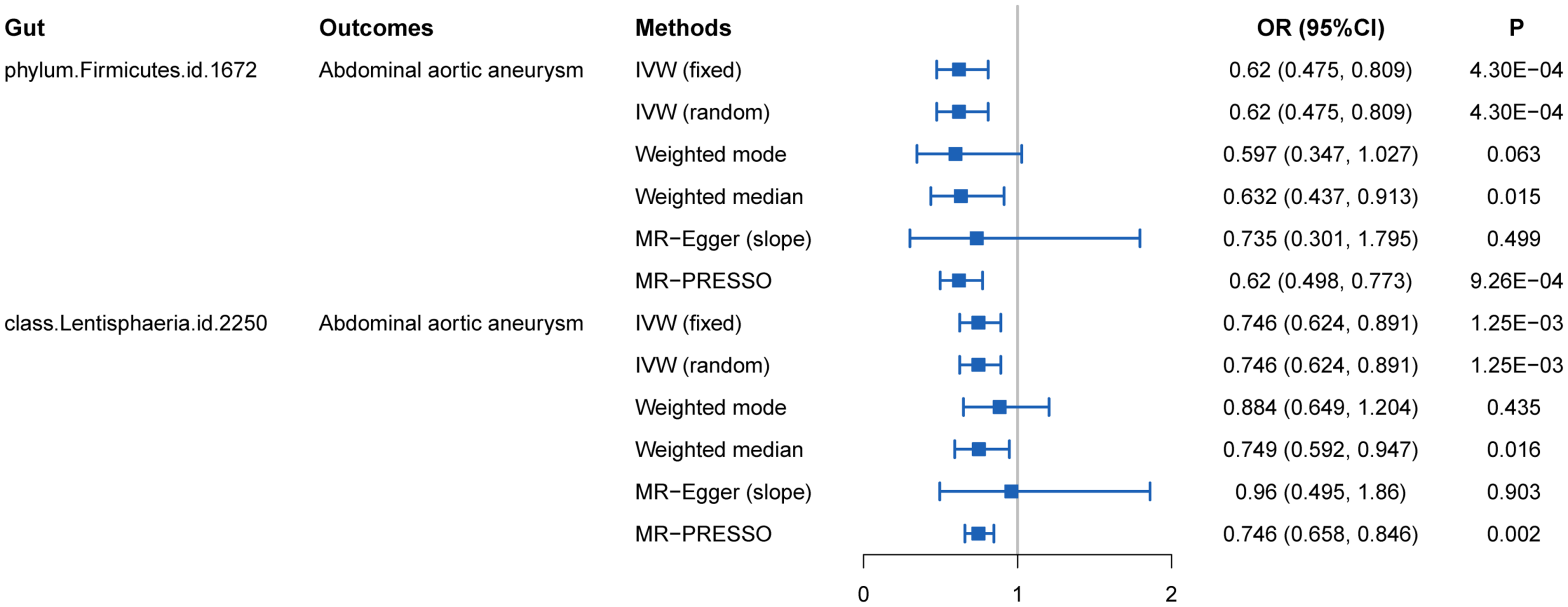
MR estimates for the causal association between gut microbiota and abdominal aortic aneurysm.

**Figure 3:**
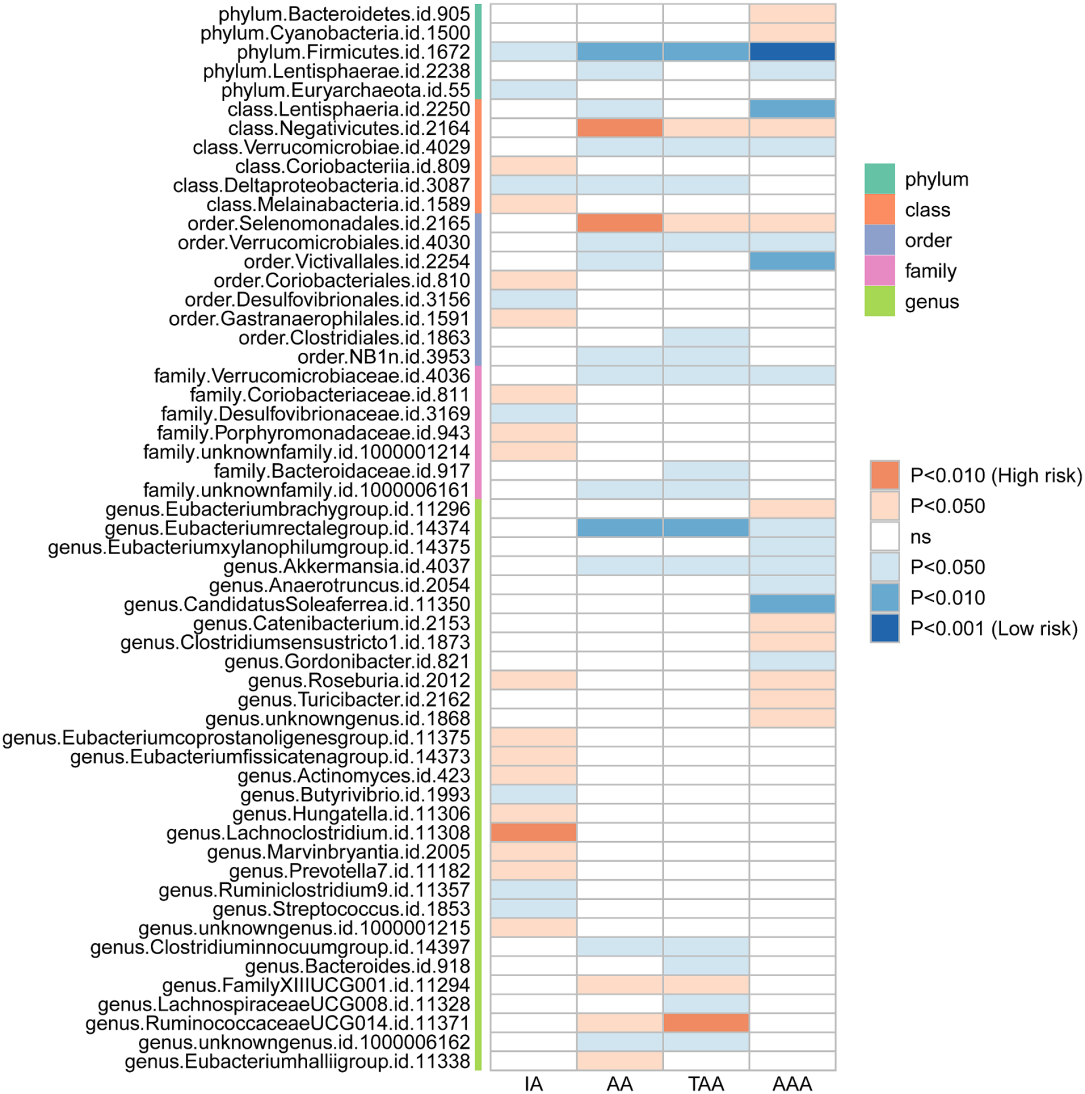
Heatmap plot of the relationship between the gut microbiomes and aneurysms.

We initially performed two-sample MR analysis on gut microbiota and aneurysms. There was no significant causal relationship between gut microbiomes and other types of aneurysms except for AAA. Of these, we found that genetically predicted phylum *Firmicutes* (OR = 0.62; 95% CI, 0.48–0.81; *p* = 4.3 × 10^−4^), class *Lentisphaeria* (OR = 0.75; 95% CI, 0.62–0.89; *p* = 1.25 × 10^−3^), and order *Victivallales* (OR = 0.75; 95% CI, 0.62–0.89; *p* = 1.25 × 10^−3^) were negatively associated with AAA risk (Table 2). Additionally, these three gut microbiomes remained causally associated with AAA after multiple-testing correction ([FDR] *q <* 0.1). Supplementary methods such as weight median and MR-PRESSO also provided consistent results with IVW, while other methods (weight mode and MR-Egger) showed the same directions, indicating the robustness of the identified SNPs (Figure 2). According to the heatmap of causality association, several gut microbiomes were found in different aneurysms (Figure 3), and the detailed information was shown in Supplementary Table S2. In suggestive significant gut microbiomes (*p <* 0.05, *q >* 0.1), genetically predicted phylum *Firmicutes*, phylum *Lentisphaera*, class *Lentisphaeria*, class *Verrucomicrobia*, class *Deltaproteobacteria*, order *Verrucomicrobiale*, family *Verrucomicrobiacea*, genus *Eubacterium rectale group*, genus *Akkermansia*, and genus *Clostridium innocuum group* were negatively associated with occurrence of different types of aneurysms, whereas genetically predicted class *Negativicutes*, order *Selenomonadales*, and genus *Roseburia* had positive causal association with different types of aneurysms.

**Table 2:** The MR results between GM taxa and aneurysm.

**Exposure**	**Outcome**	**Methods**	**OR (95%CI)**	**Pval**	**Heterogeneity**		**Horizontal pleiotropy**				**F-statistic (min)**
					**Cochran’s Q**	**Pval**	**PRESSO global test**	**Pval**	**Egger (intercept)**	**Pval**	
Phylum.Firmicutes.id.1672	AAA	IVW (fixed)	0.620 (0.475, 0.809)	4.3 × 10^−4^	8.872	0.783	10.366	0.800	−0.012	0.695	19.587
		Weighted mode	0.597 (0.347, 1.027)	0.063							
		Weighted median	0.632 (0.437, 0.913)	0.015							
		MR-Egger (slope)	0.735 (0.301, 1.795)	0.499							
		MR-PRESSO	0.620 (0.498, 0.773)	9.26 × 10^−4^							
Class.Lentisphaeria.id.2250	AAA	IVW (fixed)	0.746 (0.624, 0.891)	1.25 × 10^−3^	3.992	0.858	5.089	0.880	−0.037	0.438	20.223
		Weighted mode	0.884 (0.649, 1.204)	0.435							
		Weighted median	0.749 (0.592, 0.947)	0.016							
		MR-Egger (slope)	0.960 (0.495, 1.860)	0.903							
		MR-PRESSO	0.746 (0.658, 0.846)	0.002							
Order.Victivallales.id.2254	AAA	IVW (fixed)	0.746 (0.624, 0.891)	1.25 × 10^−3^	3.992	0.858	5.089	0.880	−0.037	0.438	20.223
		Weighted mode	0.884 (0.649, 1.204)	0.435							
		Weighted median	0.749 (0.592, 0.947)	0.016							
		MR-Egger (slope)	0.960 (0.495, 1.860)	0.903							
		MR-PRESSO	0.746 (0.658, 0.846)	0.002							

AAA, abdominal aortic aneurysm.

The outcomes of reverse MR are shown in Table 3. There was no significant causality association using the IVW method, suggesting there is no evidence of a causal effect on the risk of AAA by identified gut microbiomes (Guo et al., 2023).

**Table 3:** The outcomes of reverse MR between GM taxa and aneurysm.

Exposure	Outcomes	Methods	Beta	Se	Down	Up	Pval
AAA	phylum.Firmicutes.id.1672	IVW (fixed)	0.019	0.042	-0.064	0.103	0.648
		IVW (random)	0.019	0.056	-0.091	0.129	0.730
AAA	class.Lentisphaeria.id.2250	IVW (fixed)	-0.106	0.084	-0.271	0.059	0.208
		IVW (random)	-0.106	0.126	-0.353	0.141	0.401
AAA	order.Victivallales.id.2254	IVW (fixed)	-0.106	0.084	-0.271	0.059	0.208
		IVW (random)	-0.106	0.126	-0.353	0.141	0.401

AAA, abdominal aortic aneurysm; IVW, inverse variance weighted.

### 3.3 Sensitive analysis

The result of sensitive analysis is also shown in Table 2. According to the result of Cochran’s Q test and the MR-PRESSO method, no heterogeneity was found and no outlier was removed. In addition, *p*-values of the MR-Egger intercept and MR-PRESSO global test were above 0.05, indicating no horizontal pleiotropy in this MR study.

Because the order *Victivallales* belongs to the class *Lentisphaeria*, we performed the following analysis on phylum *Firmicutes* and class *Lentisphaeria*. In addition, the scatter plot of phylum *Firmicutes* and class *Lentisphaeria* also showed that there was no interference between associations from outliers (Supplementary Figure S1). Moreover, each method demonstrated the same direction of slope, suggesting that identified gut microbiomes were protective factors for aneurysm. According to the funnel plot, no bias was found because the estimated values were symmetrically distributed (Supplementary Figure S1). In the leave-one-out test, there was no change in risk of aneurysm as genetically predicted, proving that the MR results were robust and not driven by a single specific SNP (Supplementary Figure S2).

### 3.4 Go enrichment and tissue-specific expression analysis

**Figure 4:**
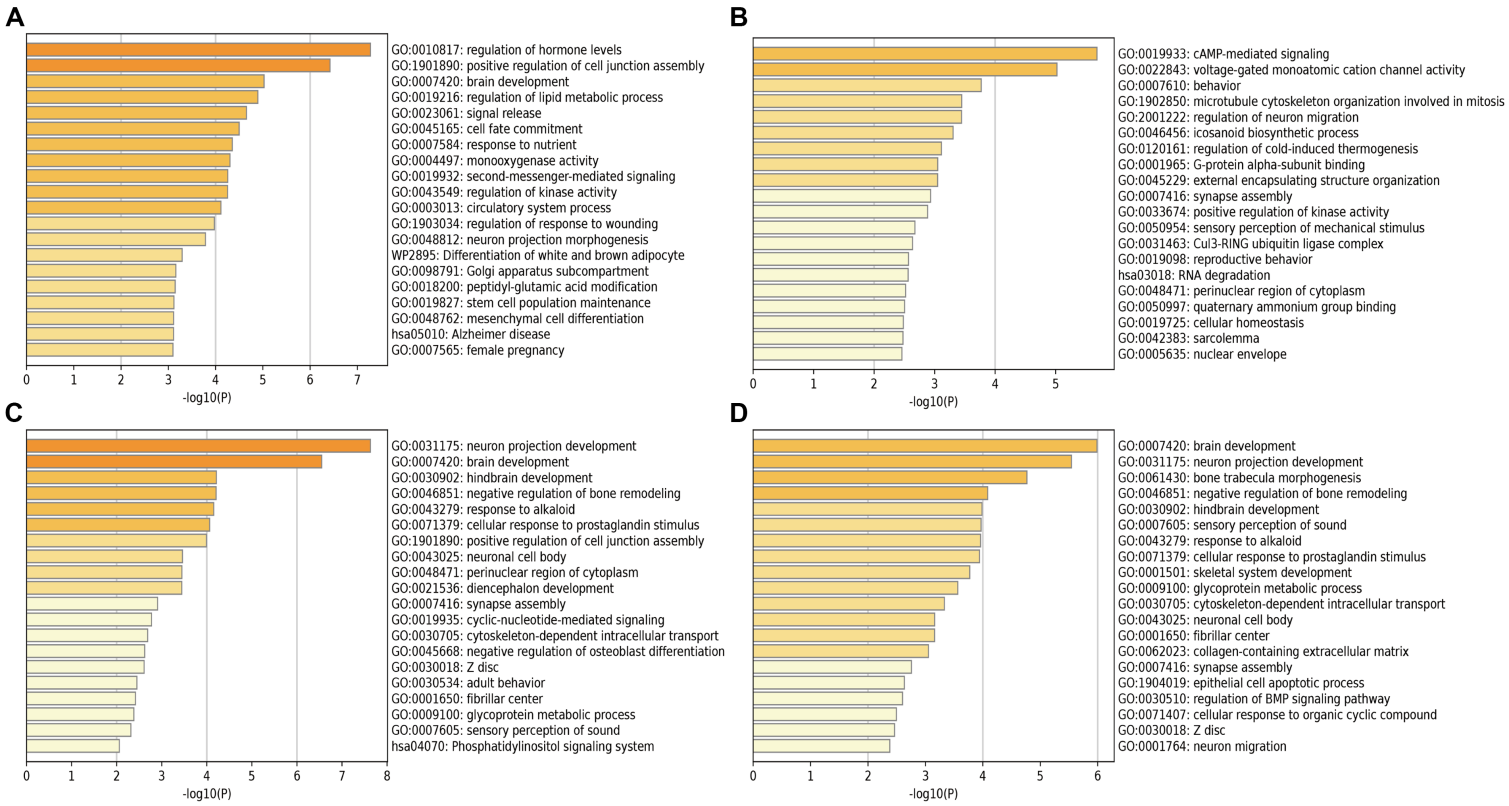
Pathway enrichment analysis of instrumental variables with suggestive causal association. **(A)** Abdominal aortic aneurysm; **(B)** Intracranial aneurysm; **(C)** Aortic aneurysm; **(D)** Thoracic aortic aneurysm.

**Figure 5:**
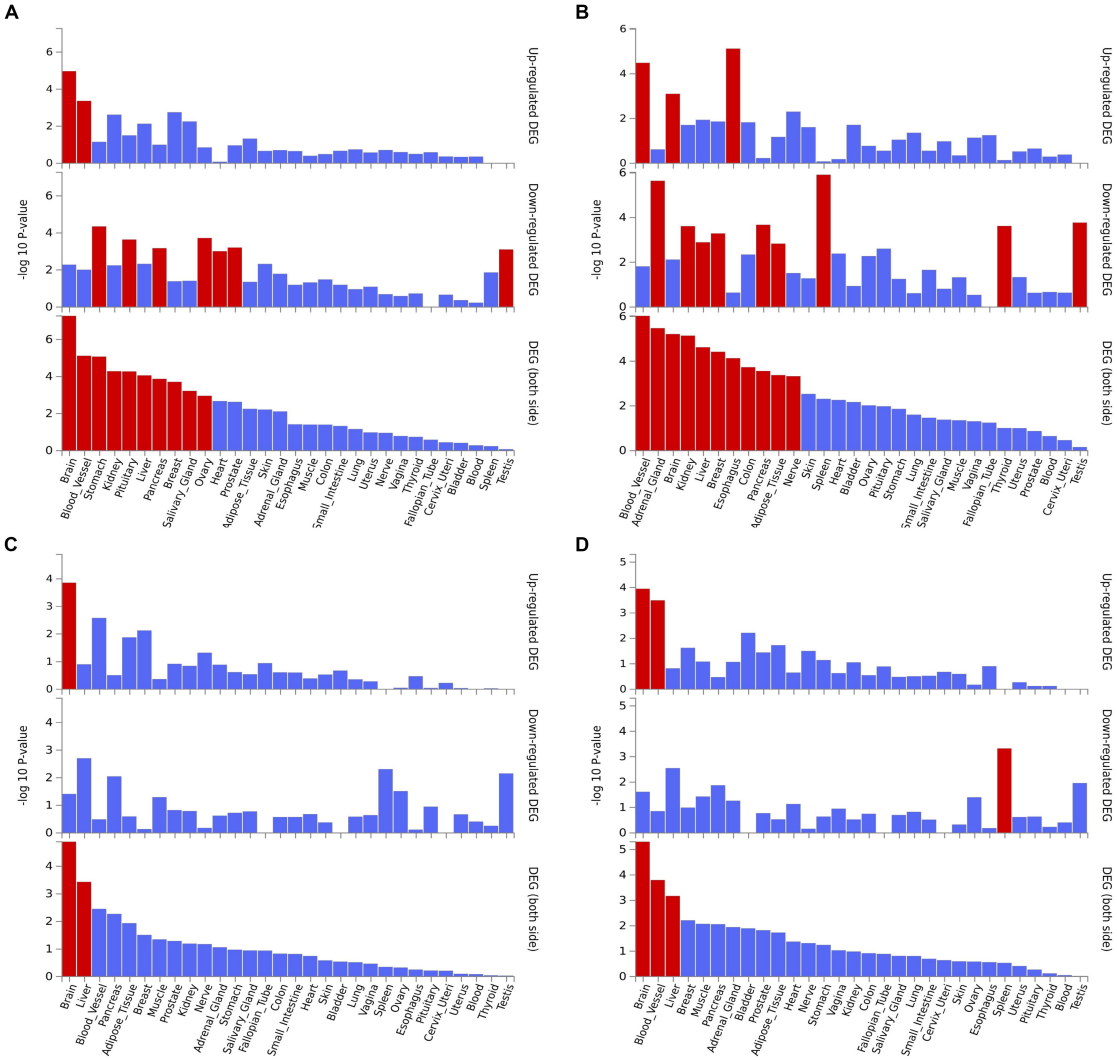
Tissue expression enrichment in GTEx tissue types. **(A)** Abdominal aortic aneurysm; **(B)** Intracranial aneurysm; **(C)** Aortic aneurysm; **(D)** Thoracic aortic aneurysm.

We performed the GO enrichment analysis on SNPs of gut microbiomes that were suggestively associated with different types of aneurysms, and the results and mapped genes are shown in Figure 4 and Supplementary Table S3. In IA, these gut microbiomes were strongly related to cAMP-mediated signaling (GO: 0019933) and voltage-gated monoatomic cation channel activity (GO: 0022843). In AAA, gut microbiomes were related to regulation of hormone levels (GO: 0010817) and positive regulation of cell junction assembly (Bromander et al., 1991). The analysis of TAA and AA demonstrated similar pathways in GO analysis, both of which were associated with neuron protection development (GO:0031175) and brain development (GO: 0007420). Tissue-specific expression analysis indicated that the gene-disease association for AAA and IA were significantly enriched in multiple tissues, such as brain, blood, kidney, liver, pancreas, and breast, whereas brain and liver demonstrated enrichment in TAA and AA (Figure 5).

## 4 Discussion

In this study, we systematically explored the causal association between gut microbiome and aneurysms using two-sample MR and found that phylum *Firmicutes*, order *Victivallales*, and class *Lentisphaeria* were negatively associated with the risk of AAA, indicating a protective regulation pattern. The phylum *Firmicutes* even showed suggestive significance as a protective factor in IA, AA, TA, and AAA. Moreover, we also found several gut microbiomes that were suggestively associated with different types of aneurysms.

The gut microbiome is a complex and dynamic collection of ecological communities mainly consisting of four bacterial phyla, including *Firmicutes*, *Actinobacteria*, *Proteobacteria*, and *Bacteroidetes*. The dysbiosis of gut microbiomes may cause metabolic, immune, and neurological diseases (Strandwitz, 2018; Xu et al., 2021). As an invisible organ, it could directly influence the vascular wall inflammatory cell infiltration manifested in an enhanced vascular wall, which is important in the progression of aneurysms (Ling et al., 2022). Our study through MR analysis explored gut microbiomes that were associated with the formation and progression of aneurysms, and shed light on the potential role of gut microbiomes in preventing the occurrence and progression of aneurysms (Ling et al., 2022).

It has been recognized that *Firmicutes* is associated with Alzheimer’s disease (Vogt et al., 2017). However, the role of it in aneurysms has not been detected. Studies have also reported that a reduction of *Firmicutes* was observed in patients with obesity and type 2 diabetes, which have been proven to be modifiable risk factors for IAs in previous Mendelian studies (Schwiertz et al., 2010; Karhunen et al., 2021; Kitten et al., 2021). In our study, *Firmicutes* was considered a significant protective factor for IAs, and the increase of *Firmicutes* may influence IAs through regulation of these modifiable risk factors. The occurrence of AAA was associated with many factors, such as atherosclerosis and inflammatory disorders (Nakayama et al., 2022). Tian et al. observed gut dysbiosis in AAA with a reduced abundance in *Firmicutes* and *Bacteroidetes* in stool samples (Tian et al., 2022). In our MR, *Firmicutes* was negatively associated with AAA and exhibited a potential association with other types of aneurysms. Therefore, the reduction of *Firmicutes* may lead to the formation of aneurysms. In addition, *Firmicutes* could produce butyrate (den Besten et al., 2013), which is a short-chain fatty acids SCFAs that has anti-inflammatory and antioxidant effects, leading to the alleviation of atherosclerosis (Kasahara et al., 2018). Tian et al. also found that butyrate could arrest the progression of AAA through inhibiting neutrophil infiltration and neutrophil extracellular traps formation (Tian et al., 2022). Moreover, butyrate could reduce the production of pro-inflammatory cytokines through regulating macrophages, thus inducing an anti-inflammatory effect (Chang et al., 2014).

Our MR also demonstrated a causal effect of the increased abundance of the phylum *Lentisphaeria* and *Victivallales* (belonging to phylum *Lentisphaeria*) as a protective effect against AAA. However, there remains a lack of studies that deal with the function or the metabolites of this taxon. Ning et al. observed the causal association of phylum *Lentisphaeria* and Parkinson’s disease (Ning et al., 2022). Ours is the first study to report the causal association of phylum *Lentisphaeria* with vascular diseases, and confirmed the protective potential of phylum *Lentisphaeria* in individuals with aneurysms, implying that it could be a novel therapeutic target for AAA. Nonetheless, its specific characteristic ought to be further explored.

Gut microbiomes that may have a suggestive association with aneurysms were also observed in our study. *Akkermansia* was negatively correlated with AA, TAA, and AAA. Another study conducted by Xie et al. observed the abundance of *Akkermansia* was significantlt different between the control and AAA groups (Xie et al., 2020). Moreover, they also found that *Akkermansia* was negatively correlated with the diameter of AAA (Xie et al., 2020). It is crucial in the treatment of AAA because it not only prevents the progression of AAA through protecting against atherosclerosis (Li et al., 2016), but the abundance of *Akkermansia* also predicts the severity of AAA. It should be noted that *Akkermansia* is the only genus of phylum *Verrucomicrobia* found in gastrointestinal samples (Geerlings et al., 2018), and both of them are suggestive protective factors in our MR analysis. Additionally, *Eubacterium rectale* was also considered a possible factor in the prevention of aneurysms in our MR analysis, and it could ameliorate the change of pathology in aneurysms through producing butyrate (Wang et al., 2021).

Although our MR analysis did not show a causal association of some gut microbiomes, they may also influence the progression of aneurysm. For AAA, Xiao et al. found an abundance of *Oscillospira*, *Coprococcus*, and *Ruminococcus gnavus* in AAA, as well as an abundance of *Akkermansia*, *Allobaculum*, and *Barnesiella ntestinihominis* in the control group (Xiao et al., 2023). Of these, *Barnesiella ntestinihominis* was considered a beneficial bacterium for host homeostasis, *Oscillospira* may have a bidirectional effect on host metabolism that could not be detected by MR analysis (Xiao et al., 2023), and *Coprococcus* was positively associated with obesity (Zhou et al., 2020). These gut microbiomes may play a role in modifiable risk factors of aneurysm or participate in the formation of aneurysms through other potential mechanisms instead of having a causal association with aneurysms. Moreover, the intricate symbiotic or antagonistic relationship among gut microbiomes should not be neglected (Tian et al., 2022).

Further investigation was also conducted on enrichment analysis of different types of aneurysms. In GO enrichment analysis, we found SNPs of gut microbiomes that were suggestively associated with IA were enriched in cAMP signaling pathway, which were probably related to the formation and rupture of IA (Du et al., 2020); while voltage-gated monoatomic cation channels, such as K^+^ and Ca^+^ channels, were reported to be associated with cerebral vasospasm after the rupture of IA (Cataldi, 2013; Koide and Wellman, 2013). In addition, the regulation of hormone levels was associated with AAA in GO enrichment analysis. Makrygiannis et al. also reported the discrepancy of AAA rupture between men and women, and the protective role of endogenous estrogens in the prevention of AAA (Makrygiannis et al., 2014). In tissue-specific expression analysis, the GWAS result of significant gut microbiomes that were enriched in IVs highly expressed in blood vessel, liver, and brain suggested that these gut microbiomes may influence gene expression in these organs, leading to the formation of aneurysms. Additionally, intestinal barrier dysfunction could cause the spread of inflammation and allow gut microbiomes or their metabolites to enter into the systemic circulation, even colonizing in aneurysms (Marques da Silva et al., 2006; Clifford and Hoffman, 2015). Systemic inflammation may also result in greater permeability of the blood–brain barrier, leading to the colonization of gut microbiomes in IAs (Nelson et al., 2021).

This study has multiple strengths. First, unlike other epidemiological studies, MR analysis is less vulnerable to confounding factors and reverse causality. Second, stringent quality control procedures and sensitivity analysis were conducted in this study to assess the robustness of the MR estimate. Third, a rigorous FDR correction was applied in the MR analysis to minimize type I errors. Fourth, further analysis on the potential pathway and tissue-specific expression was also conducted to investigate the mechanisms underlying the links between GM and different types of aneurysms. However, study’s limitations should also be noted. First, since our MR analysis used pooled estimates instead of raw data, we did not conduct subgroup analysis of different sizes of aneurysms or explore non-linear relations. Second, our study analyzed only populations in Europe. Thus, we should interpret the results with caution because they may not be applicable for other ethnic groups. Third, gut microbiomes were analyzed only at the genus level instead of a more specialized level such as strain levels or species. Fourth, we could not exclude the effect of gene–environment interactions on outcome as it has been acknowledged that environmental factors are relatively important in the pathophysiology of IA (Shikata et al., 2019). Last, our results were based on MR estimation without considering the evidence from basic or clinical researches. Thus, in order to figure out the potential mechanism of the influence of GM on aneurysms, further investigations that comprehensively assess the relationship between them through multi-omics perspective were required.

## 5 Conclusion

We provide evidence supporting the causal effect of the abundance of specific gut microbiomes on the risk of aneurysms. Of these, phylum *Firmicutes*, class *Lentisphaeria*, and order *Victivallales* have a causal association with AAA. The decreased abundance of these gut microbiomes may lead to higher risk of AAA. However, the mechanism of the latter two in AAA should be further explored. Meanwhile, several gut microbiomes that were suggestively associated with different types of aneurysms should be explored as potential targets in aneurysm prevention.

## Data availability statement

The original contributions presented in the study are included in the article/Supplementary material, further inquiries can be directed to the corresponding authors.

## Author contributions

YQ: Conceptualization, Writing – original draft, Investigation. YH: Conceptualization, Writing – original draft, Investigation. XW: Conceptualization, Writing – original draft, Investigation. MW: Data curation, Writing – original draft. ZY: Data curation, Writing – original draft. MX: Methodology, Software, Writing – original draft. AD: Methodology, Software, Writing – original draft. CM: Conceptualization, Validation, Writing – review & editing. KS: Conceptualization, Resources, Supervision, Writing – review & editing. ZW: Conceptualization, Resources, Supervision, Writing – review & editing.
