# Supplementary material for: Causal association between gut microbiomes and different types of aneurysms: a Mendelian randomization study
Source: Front Microbiol. 2024 Apr 10;15:1267888. doi: 10.3389/fmicb.2024.1267888 (PMC11039950; doi:10.3389/fmicb.2024.1267888)
Supplement: Supplementary file 1 [file Table_1.docx]

Supplementary Material

**Causal Association Between Gut Microbiomes and Different Types of Aneurysms: A Mendelian Randomization Study**

Youjia Qiu ^1, #^, Yucheng Hou ^2, #^, Xingzhou Wei ^3, #^, Menghan Wang ^3^, Ziqian Yin ^1^, Minjia Xie ^1^, Aojie Duan ^1^, Chao Ma ^1, *^, Ke Si ^2, *^, Zhong Wang ^1, *^

^a^ Department of Neurosurgery & Brain and Nerve Research Laboratory, The First Affiliated Hospital of Soochow University, Suzhou, Jiangsu Province, 215006, China

^b^ Department of Cardiovascular Surgery, The First Affiliated Hospital of Soochow University, Suzhou, Jiangsu Province, 215006, China

^c^ Suzhou Medical College of Soochow University, Suzhou, Jiangsu Province, 215002, China

^#^ Youjia Qiu and Yucheng Hou contribute equally to this work.

*** Correspondence:**Zhong Wang, Department of Neurosurgery & Brain and Nerve Research Laboratory, The First Affiliated Hospital of Soochow University, Suzhou, Jiangsu Province, 215006, China. Email address: [wangzhong761@163.com](mailto:wangzhong761@163.com).

Ke Si, Department of Cardiovascular Surgery, The First Affiliated Hospital of Soochow University, Suzhou, Jiangsu Province, 215006, China. Email address: [sike@suda.edu.cn](mailto:sike@suda.edu.cn)

Chao Ma, Department of Neurosurgery & Brain and Nerve Research Laboratory, The First Affiliated Hospital of Soochow University, Suzhou, Jiangsu Province, 215006, China. Email address: machaoss@163.com

## Supplementary Figures

Figure S1. Scatter plots and funnel plot of phylum *Firmicutes*, and class *Lentisphaeria*.


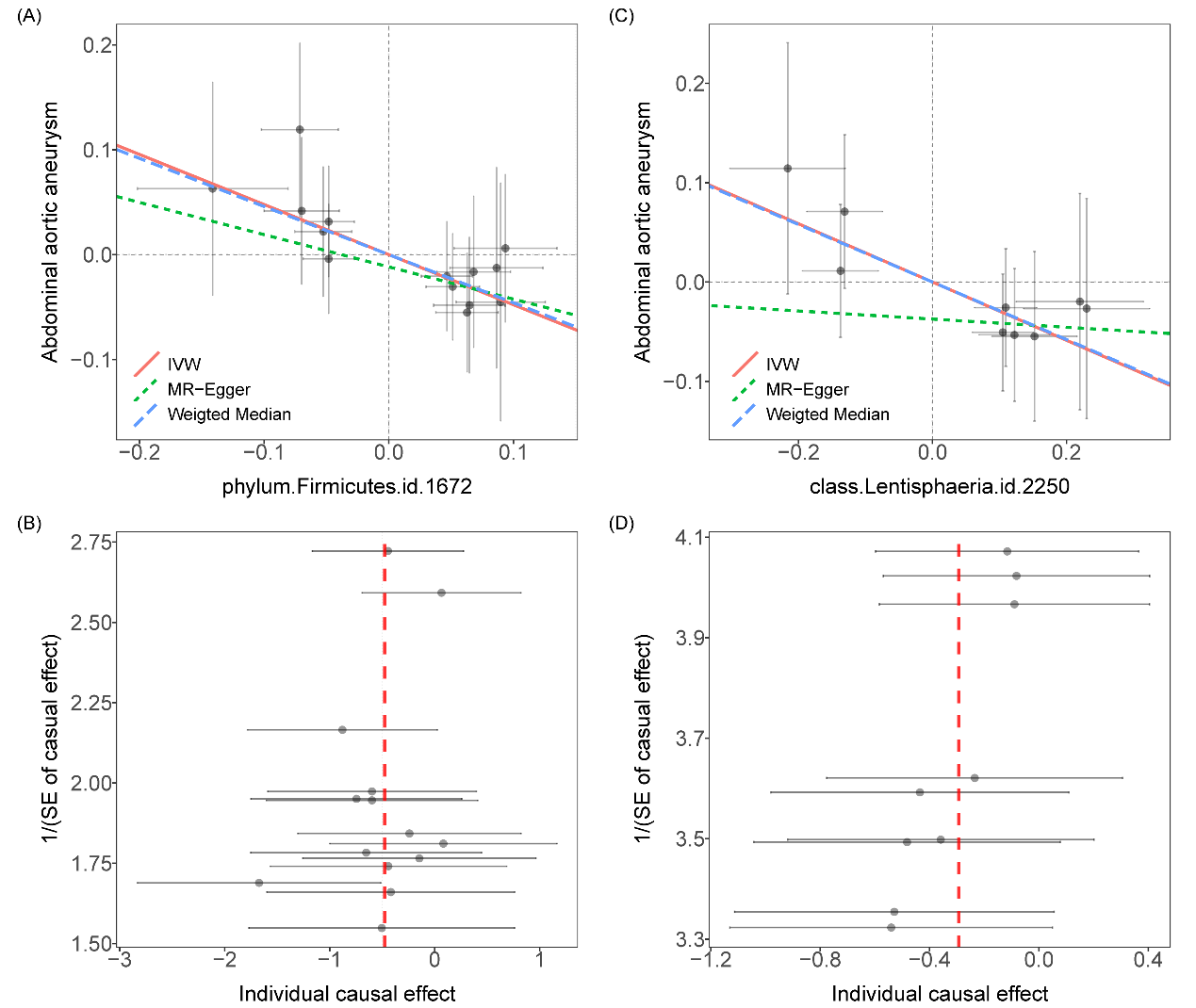


Figure S2. Leave-one-out plots for the causal association between gut microbiota and abdominal aortic aneurysm (AAA). (A) phylum *Firmicutes*; (B) class *Lentisphaeria*.


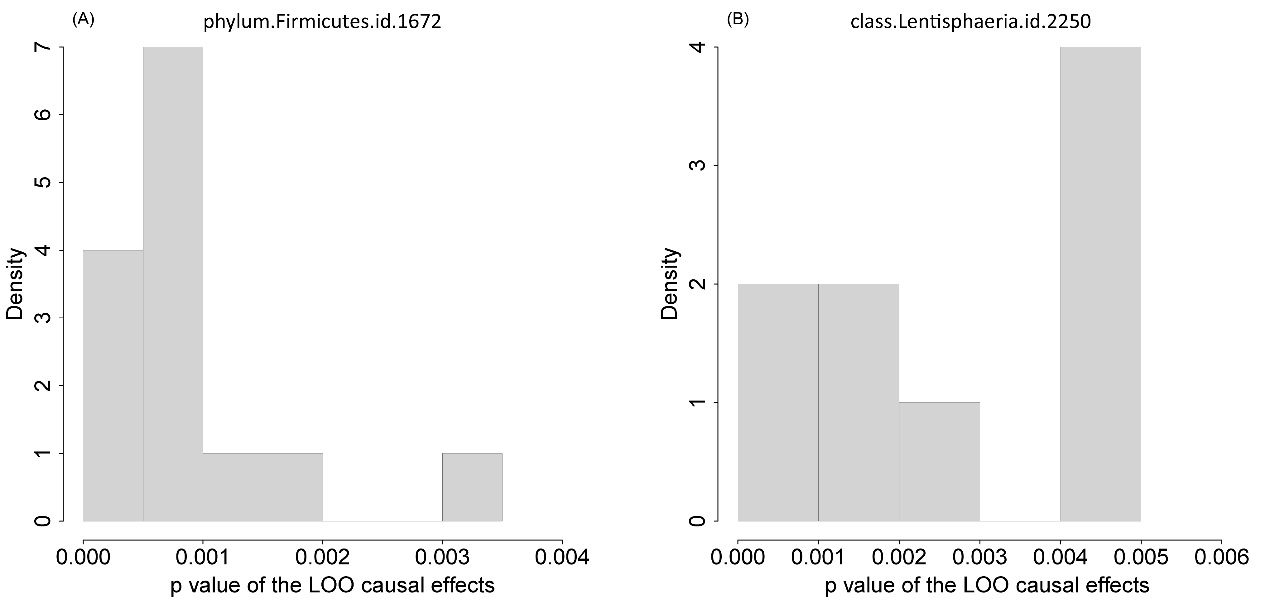


**
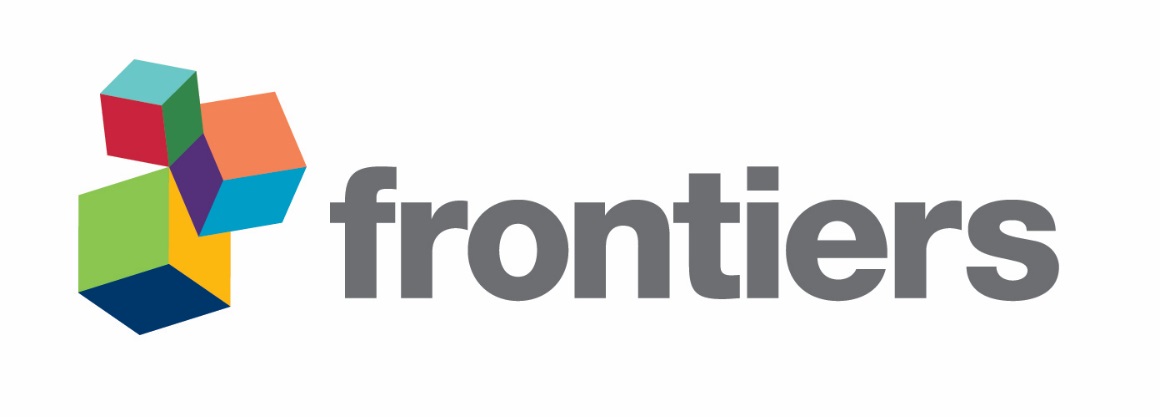
**

**Supplementary Figure 1.** Scatter plots and funnel plot of phylum *Firmicutes*, and class *Lentisphaeria*.

**Supplementary Figure 2**. Leave-one-out plots for the causal association between gut microbiota and abdominal aortic aneurysm (AAA). (A) phylum *Firmicutes*; (B) class *Lentisphaeria*.
